# High-resolution open-top axially swept light sheet microscopy

**DOI:** 10.1186/s12915-023-01747-3

**Published:** 2023-11-08

**Authors:** Soohyun Park, Myeongsu Na, Sunghoe Chang, Ki Hean Kim

**Affiliations:** 1 Department of Mechanical Engineering, Pohang University of Science and Technology, 77 Cheongam-Ro, Nam-gu, Pohang, Gyeongbuk 37673 Republic of Korea; 2 Department of Research and Development Center, Crayon Technologies, 19 Sanmaru-ro, Guri, Gyeonggi-do 11901 Republic of Korea; 3 Department of Physiology and Biomedical Sciences, Seoul National University College of Medicine, 103 Daehak-ro, Jongno-gu, Seoul, 03080 Republic of Korea; 4 Neuroscience Research Institute, Seoul National University College of Medicine, 103 Daehak-ro, Jongno-gu, Seoul, 03080 Republic of Korea

**Keywords:** Light sheet microscopy, Open-top, Axial swept, Aberration correction, Optically cleared tissue

## Abstract

**Background:**

Open-top light-sheet microscopy (OT-LSM) is a specialized microscopic technique for the high-throughput cellular imaging of optically cleared, large-sized specimens, such as the brain. Despite the development of various OT-LSM techniques, achieving submicron resolution in all dimensions remains.

**Results:**

We developed a high-resolution open-top axially swept LSM (HR-OTAS-LSM) for high-throughput and high-resolution imaging in all dimensions. High axial and lateral resolutions were achieved by using an aberration-corrected axially swept excitation light sheet in the illumination arm and a high numerical aperture (NA) immersion objective lens in the imaging arm, respectively. The high-resolution, high-throughput visualization of neuronal networks in mouse brain and retina specimens validated the performance of HR-OTAS-LSM.

**Conclusions:**

The proposed HR-OTAS-LSM method represents a significant advancement in the high-resolution mapping of cellular networks in biological systems such as the brain and retina.

**Supplementary Information:**

The online version contains supplementary material available at 10.1186/s12915-023-01747-3.

## Background

Visualization of cellular networks in the intact brain is important for understanding its structure and function. It would help to decipher the functional structure of healthy brains and the mechanisms of neurological pathologies. Recent advances in optical clearing, molecular labeling, and optical microscopy techniques have facilitated the network visualization. Owing to its unrivaled speed and low photobleaching, light-sheet microscopy (LSM) has emerged as a preferred method for high-resolution volumetric imaging of cleared tissue. Open-top LSM (OT-LSM) is a specialized LSM for the high-throughput imaging of large tissue without size restriction [1, 2], and it enabled the imaging of optically cleared tissue specimens such as brains and human cancers for neuronal network study and nondestructive 3D histology, respectively [3–6]. Notably, various OT-LSM configurations exist. Conventional OT-LSMs had separate illumination and imaging arms that were orthogonal to each other and angled with respect to the sample surface, and they are called orthogonal dual objective (ODO) systems. Because light entered the sample with an angle, additional interfacing devices such as a liquid prism [1], a solid immersion lens (SIL) [3, 7–9], and a solid immersion meniscus lens (SIMlens) [10] were used to minimize optical aberration. ODO OT-LSMs had the advantage of utilizing full numerical aperture (NA) of the imaging objective lens for high resolution, while having the disadvantages of limited working distance, high sensitivity to aberration, and high complexity. Recently, LSMs using single-objective lenses for both the oblique light sheet illumination and emission light collection were developed, and they are called single-objective (SO) systems. The oblique imaging plane in the sample was relayed to a remote focus and then re-imaged by an additional imaging setup in the proper orientation. The scanning version was capable of high-speed volumetric imaging and was primarily used for live small animal studies [11, 12]. They had the advantages of low sensitivity to optical aberration and utilizing the full working distance of the objective lens. However, they had relatively poor axial resolutions of more than 1 µm due to the trade-off of the cone angles of illumination and emission light within the limited NA of the single objective lens and their small crossing angle of less than 90° [13]. A non-orthogonal dual-objective (NODO) OT-LSM, combining the advantages of both dual- and single-objective structures, was developed. The system had an obliquely oriented illumination arm and a normally oriented imaging arm. However, NODO OT-LSM still had a relatively poor axial image resolution of 2.9 µm due to the small crossing angle [14]. Overall, these OT-LSM configurations have their own advantages and disadvantages, and conventional ODO OT-LSMs are still the best for high-resolution imaging. Various high-resolution OT-LSM methods were developed. However, the resolution improvement was mostly in the lateral resolution. The axial resolution remained poor because the excitation light sheet was weakly focused to illuminate the entire imaging field of view (FOV). Axially swept LSMs (AS-LSMs) were developed for high axial resolutions by axially sweeping a tightly focused illumination light sheet across the FOV and collecting emission light in the sweeping focus with a camera in rolling shutter mode [15–18]. Although open-top AS-LSM (OTAS-LSM) was developed previously, it still had relatively poor resolutions of approximately 1.5 µm by using low NA air objectives. High-resolution OT-LSMs with submicron resolutions in all dimensions remain unachieved.

This study presents a high-resolution open-top axially swept light sheet microscopy (HR-OTAS-LSM) in the ODO configuration for submicron-resolution high-throughput imaging. The high axial resolution was achieved by using a deformable mirror (DM) for both the axial sweeping of the excitation light sheet and correcting the system aberration, and the high lateral resolution was realized by employing a high-NA immersion objective lens in the imaging arm. After design, implementation, and characterization, the proposed system was applied to optically cleared mouse brain and transparent mouse retina specimens for performance verification.

## Results

### System design and development

**Fig. 1:**
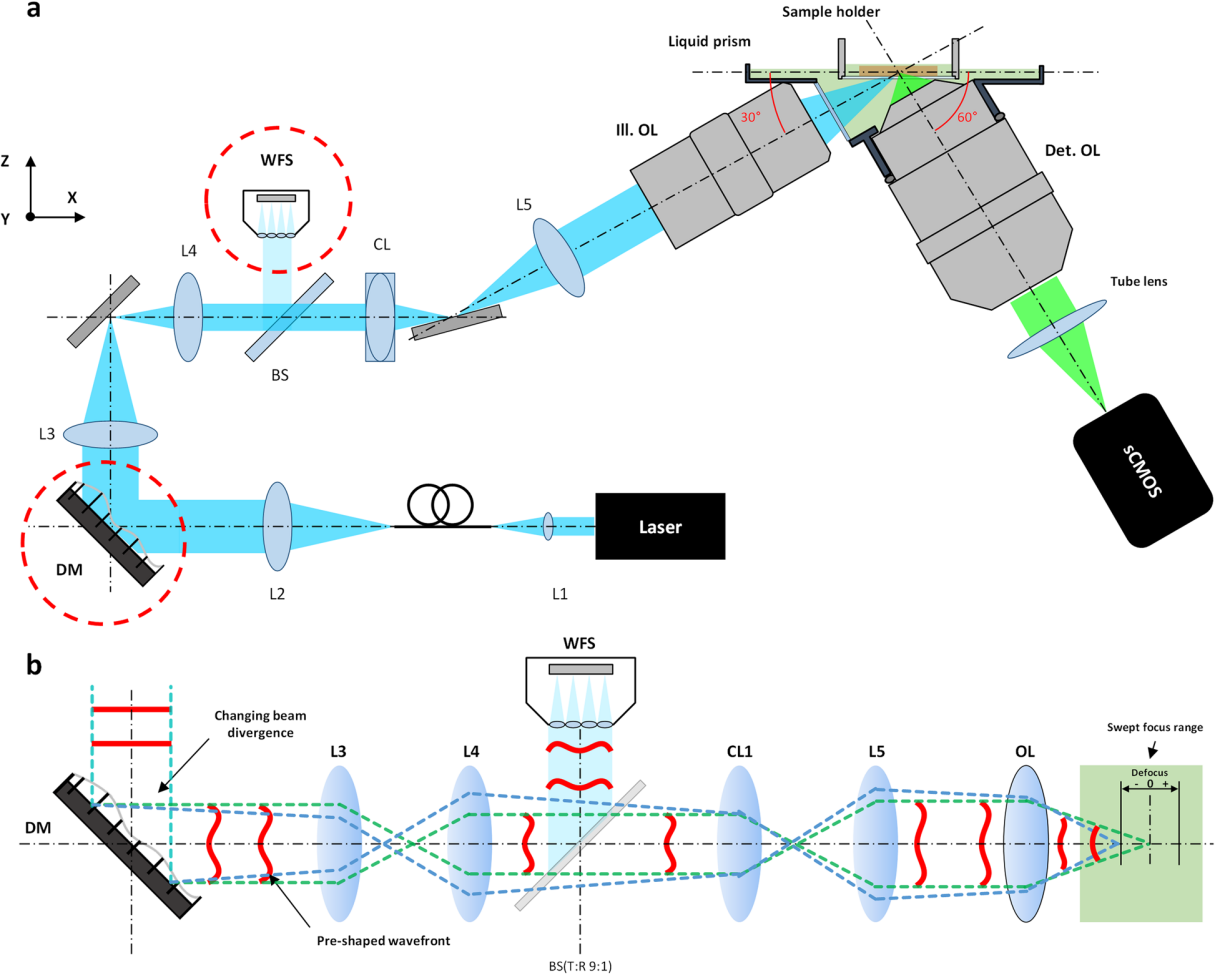
Schematics of HR-OTAS-LSM. **a** An overall schematic of HR-OTAS-LSM. **b** A schematic showing the detailed action of the DM and the WFS in the illumination arm. DM, deformable mirror; WFS, wavefront sensor; OL, objective lens; CL, cylindrical lens

Figure 1 presents the schematics of the HR-OTAS-LSM system. This system is a dual-objective lens type in the orthogonal arrangement. A long working distance air objective lens (MY10X-803, NA 0.28, Mitutoyo) and a multi-immersion objective lens (CFI90 20XC Glyc, NA 1.0, Nikon) were used in the illumination and imaging arms, respectively. A custom liquid prism was used as the interfacing device. The illumination and imaging objectives were angled at 30° and 60° with respect to the sample surface, respectively. It was to increase the usable working distance of the imaging objective lens and the imaging depth. In the illumination arm, excitation light (488 nm) from a continuous wave (CW) laser (Sapphire 488 LP-100, Coherent) was delivered to the system through a single-mode fiber (SM1FC, Thorlabs; P1-460Y-FC-1, Thorlabs). Excitation light from the fiber was collimated by a lens (L2, AC254-050-A, Thorlabs), and the illumination beam was then reflected on a DM (DMH40-P01, Thorlabs). The DM was both to change the beam divergence for the axial sweeping of the illumination light sheet in the sample and to generate a counter aberration for the correction of system aberration. The illumination beam passed through a lens pair (L3 and L4, AC254-100-A and AC254-50-A, Thorlabs) and then through a beam splitter (BS, BSN10R, Thorlabs). The beam splitter reflected 10% of the illumination beam toward a wavefront sensor (WFS, WFS-14AR, Thorlabs) for wavefront measurement. The transmitted beam was converted to a light sheet by a cylindrical lens (CL, LJ1695RM-A, Thorlabs), and the light sheet was relayed to the sample by a combination of a lens (L5, AC254-100-A, Thorlabs) and the objective lens. Illumination light from the air objective lens entered the sample through the liquid prism, which held a refractive index (RI) matching solution (C match, 1.46 RI, Crayon Technologies, Korea) and formed a planar interface for the normal incidence of illumination light onto the RI matching solution. Although the prism removed off-axis optical aberrations in the illumination light sheet, it left on-axis spherical aberration (SA) caused by the RI mismatch between air and the RI matching solution in the path of illumination light. The system aberration was corrected by the DM. After passing through the RI matching solution in the liquid prism and a quartz coverslip at the bottom of a sample holder, the illumination light sheet was focused within the sample. The sample holder was connected to a motorized XYZ stage (MS-2000 XYZ, ASI) for sample translation in both the lateral and axial directions. Emission light generated in the sample was collected by the imaging objective lens and immersed in the liquid chamber. After the objective lens, emission light passed through an emission filter (ET525LP, Chroma) and a camera lens (A17 AF 70-300 mm F4-5.6 Di LD MACRO 1:2, Tamron) and was then collected at a sCMOS camera (pco.edge 4.2, PCO). The camera worked in rolling shutter mode, where the movement of the rolling shutter was synchronized with that of the axially sweeping excitation light sheet for confocal detection. The collected image data was transferred to a data acquisition computer via a frame grabber (Firebird camera link frame grabber, Active Silicon). The selective planar imaging of the samples, angled at 30° with respect to the sample surface, was conducted sequentially with the stepwise lateral translation of the sample. The imaging FOV was approximately 460 µm × 460 µm with 2048 × 2048 pixels, determined by the × 20 imaging objective and the camera. Owing to the inclined imaging plane, this FOV covered 230 µm in depth. The step size for lateral translation was 0.2 µm, which was half the designed lateral resolution. The maximum imaging speed was 50 fps in full-frame acquisition, limited by the camera. The camera exposure time was approximately 20 µs per line. Volumetric imaging at different depths was conducted with the axial sample translation up to 1.5 mm from the surface.

### System characterization

**Fig. 2:**
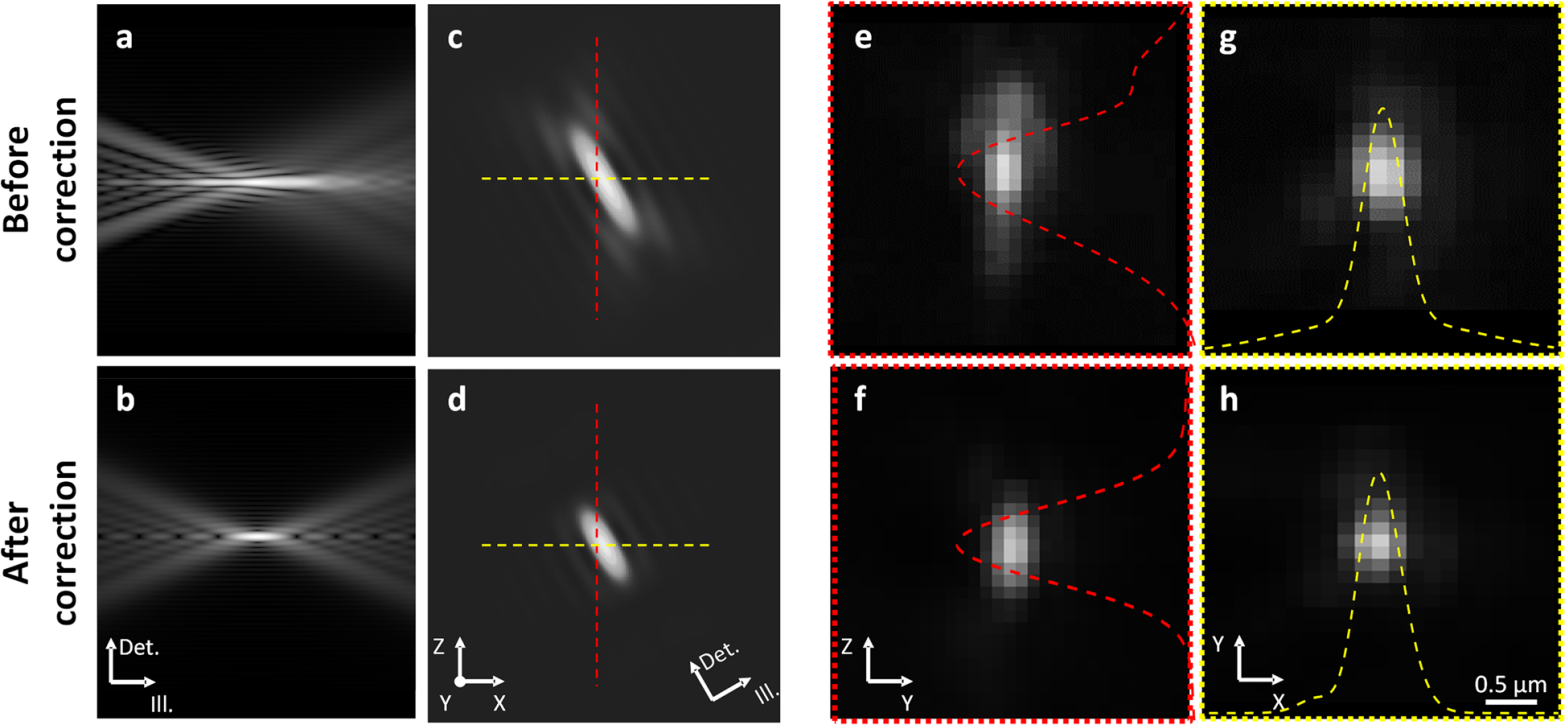
Resolution analysis after aberration correction. Results before and after the correction are shown in the upper and lower rows, respectively. **a**, **b** Simulated illumination light sheets. **c**, **d** Simulated system point spread functions (PSFs). **e**–**h** Measured PSFs in the axial and lateral directions

Figure 2 shows the resolution analysis of HR-OTAS-LSM by both simulation and measurement. For the measurement, fluorescent microspheres (0.2 µm in D), immobilized in agarose gel, were imaged as point sources. The simulation and measurement results before and after aberration correction are presented in the upper and lower rows, respectively. Before the correction, the simulation results showed a broadened focus of the excitation light sheet (Fig. 2a). The system’s point spread function (PSF), which was the product of illumination and imaging PSFs, showed the axial resolution degradation in Fig. 2c. The measured intensity profiles of microsphere images in the *y*–*z* and *x*–*y* planes are shown in Fig. 2e–h, respectively. The intensity profiles in the *y*–*z* plane showed a relatively high background around the peak before the correction, and they became narrow with less background after the correction. The full width at half maximum (FWHM) was measured to be 0.4 ± 0.1 µm in the *x*–*y* plane and 0.9 ± 0.2 µm in the z-axis, respectively. These were within approximately 150% of the simulated or theoretical values of 0.28 µm and 0.71 µm, respectively.

### HR-OTAS-LSM imaging of optically cleared mouse brain

**Fig. 3:**
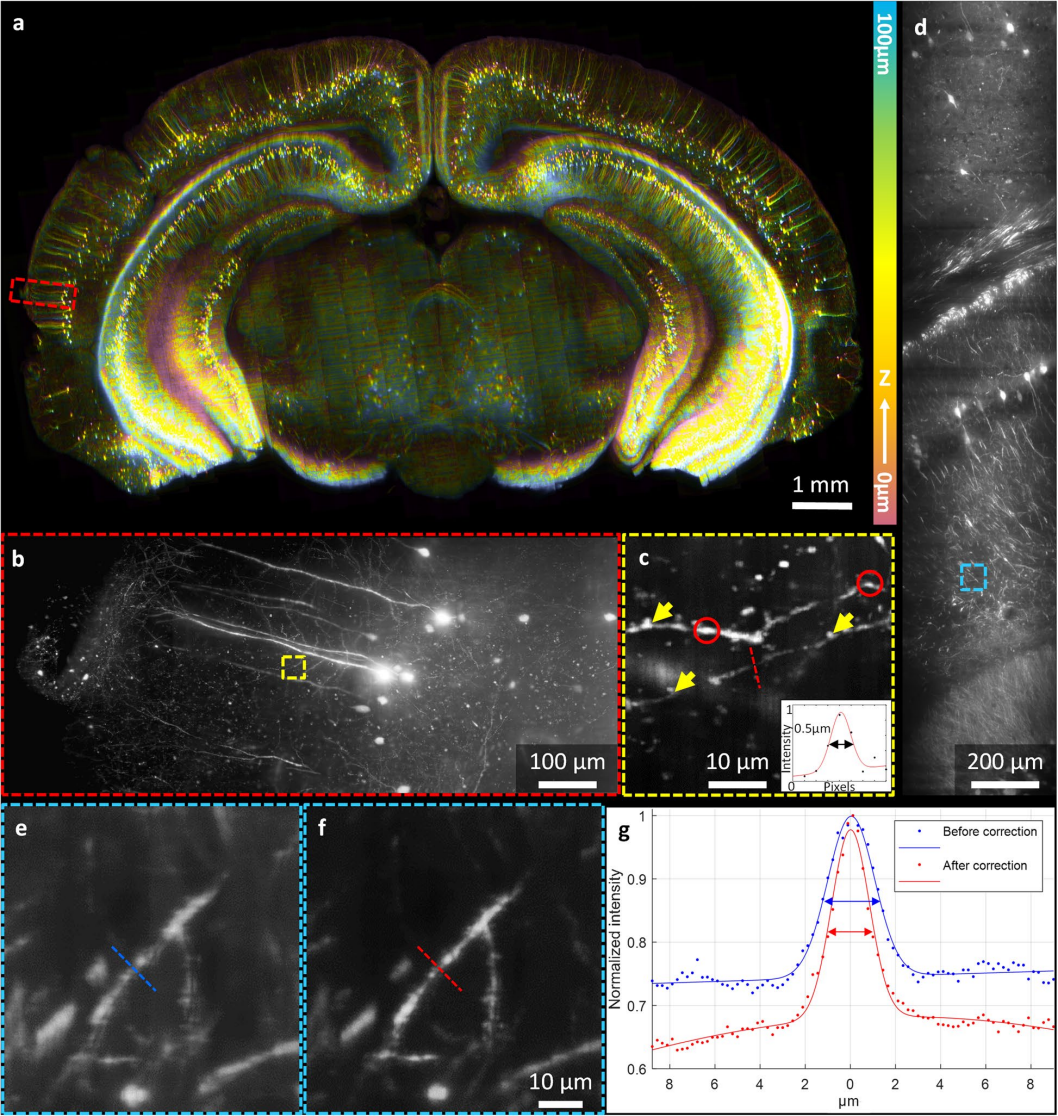
HR-OTAS-LSM images of an optically cleared Thy1-eYFP mouse brain. **a** A large sectional image of the brain slice with depth color coding from 0 to 100 µm. **b**, **c** Magnified images in two different steps to demonstrate sub-micron resolution imaging of a single dendrite. **d** A magnified image in a cerebral cortex region. **e**, **f** Dendrite images without (**e**) and with (**f**) correction. **g** Intensity profiles of a dendrite in **e** and **f**

Figure 3 shows the HR-OTAS-LSM images of an optically cleared mouse brain slice. A large FOV image of the entire brain slice and two magnified images at different scales are shown. The large FOV image had 13 mm × 7.5 mm in FOV and was depth color-coded down to 100 µm deep from the surface. The magnified images were presented as the maximum intensity projection (MIP) of 3D images with a 20-µm depth range. The large FOV image showed a gross neuronal network in the mouse brain: various neuron arrangements at different densities, depending on regions and their inter-regional connections. The magnified images showed detailed cellular information. Two magnified images in the cerebral cortex showed detailed neuron distribution and morphology (Fig. 3b, d). Cortical pyramidal neurons with apical dendrites extended toward the cortical surface. An ROI was selected over a sparsely distributed neuron in Fig. 3b and was magnified further to show details in Fig. 3c. Dendrites of a single neuron were visualized with individual spines (yellow arrow) and varicosities (red circle). The intensity profile across a thin dendrite was approximately 0.5 μm in FWHM. The effects of aberration correction were analyzed in the mouse brain images. Magnified images in a cerebral cortex region both without and with aberration correction were presented in Fig. 3e, f. While the image without correction showed relatively hazy structures, the one with correction showed the same structures in higher contrast and without a hazy background. The major effect of aberration correction was the improvement of image contrast (Fig. 3g).

**Fig. 4:**
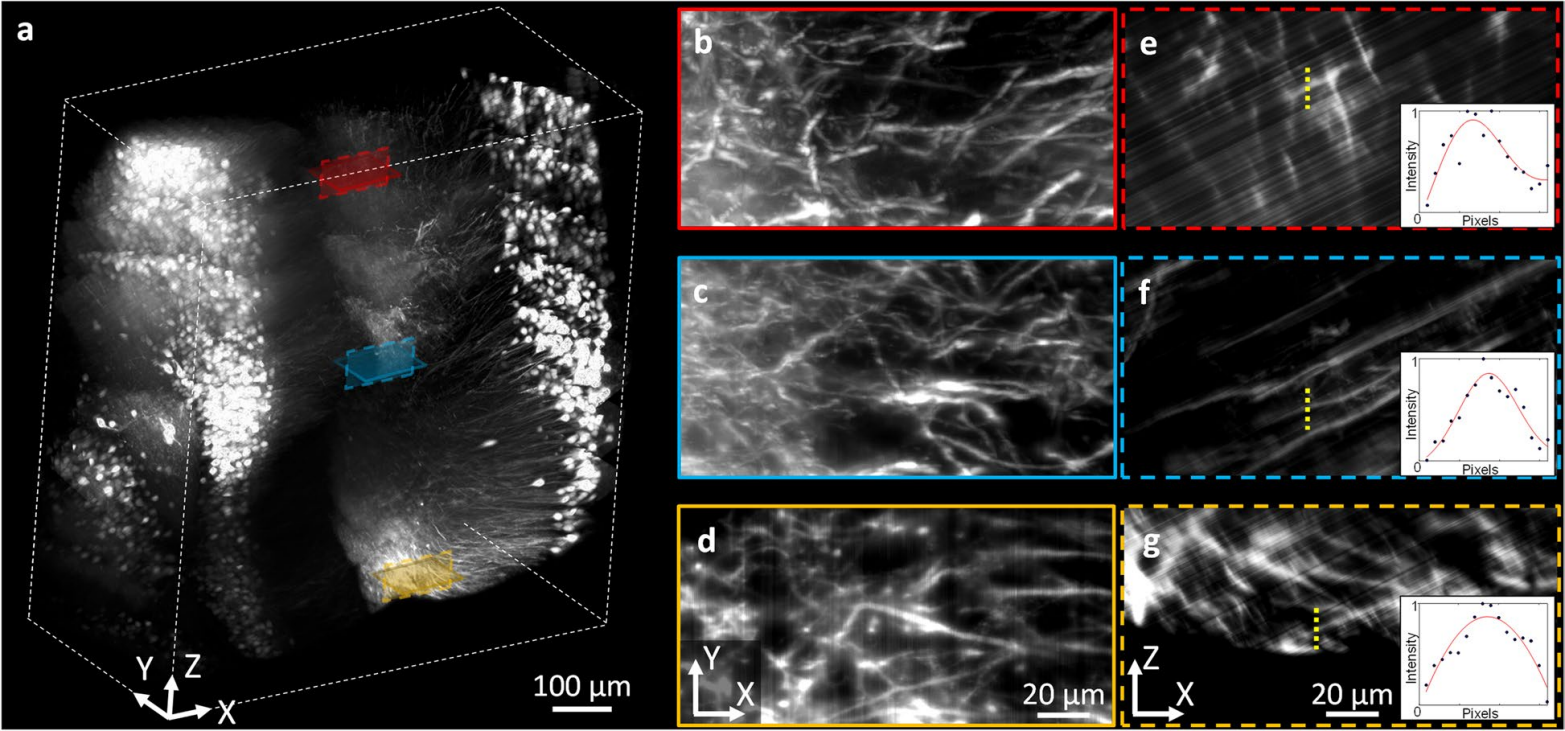
A volumetric image and magnified 2D MIP images in the cerebral cortex of an optically cleared Thy1-eYFP mouse brain. **a** A 3D rendered image up to 1.5 mm depth. **b**–**d** Magnified images in the *x*–*y* plane at different depths. **e**–**g** Magnified images in the *x*–*z* planes at different depths

Figure 4 depicts a volumetric image of the cerebral cortex in a 1.5-mm depth range. The image was 0.45 mm × 2 mm in the *x*–*y* plane. Neurons aligned in the *x* direction were uniformly distributed throughout the depth range. A series of 2D images in the *x*–*y* and *x*–*z* planes at various depths of 0 mm (Fig. 4d, g), 0.8 mm (Fig. 4c, f), and 1.5 mm (Fig. 4b, e) from the bottom surface are presented. These 2D images were generated as MIPs of 3D images with a 20-µm thickness range to visualize the morphology of neuronal structures. These 2D MIP images at different depths showed that the image resolution did not change in the imaged depth range.

### Comparison of HR-OTAS-LSM, confocal microscopy, and low-resolution OTAS-LSM in optically cleared mouse brain

**Fig. 5:**
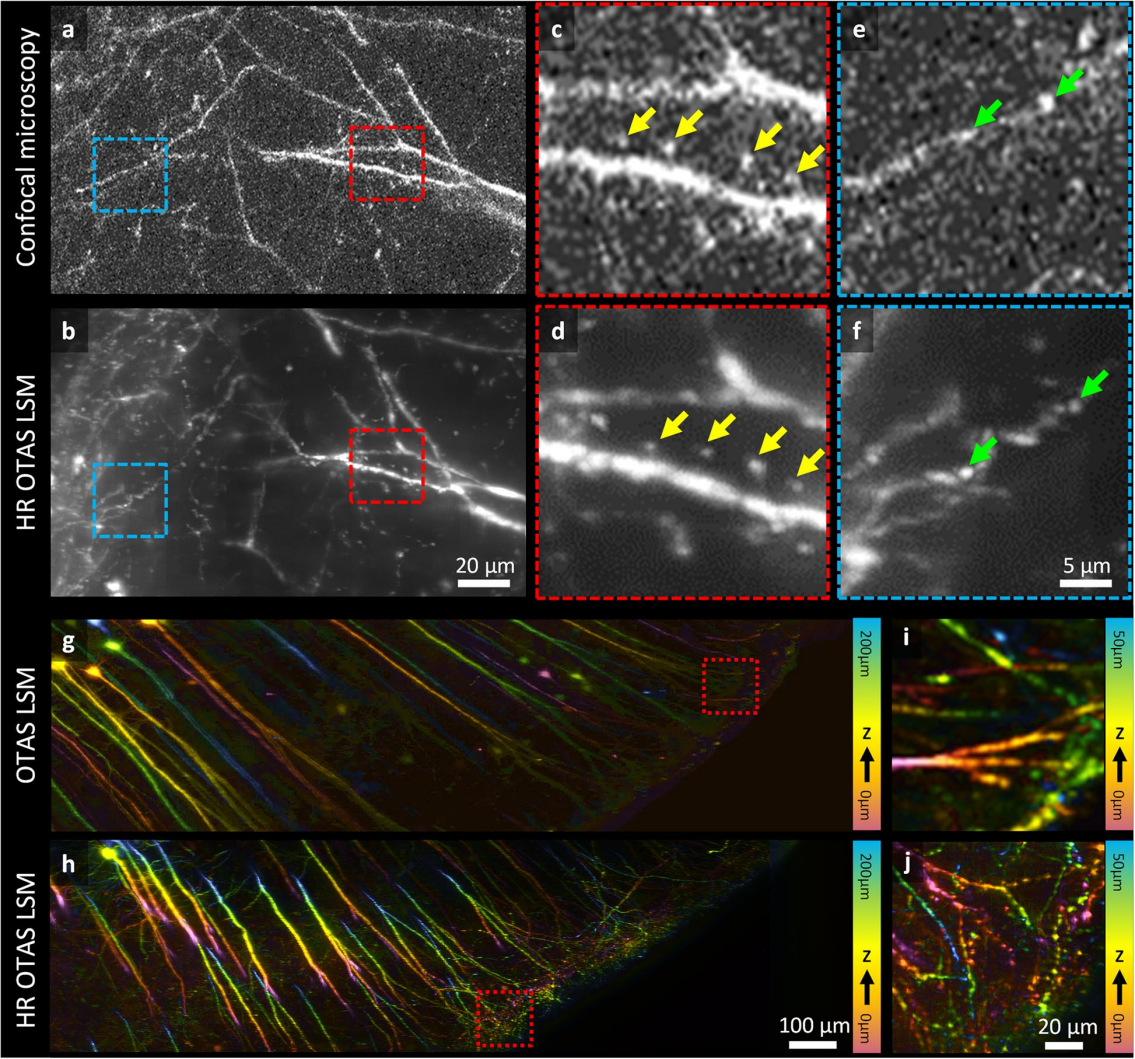
Comparison of HR-OTAS-LSM with conventional confocal microscopy (CM) and low-resolution (LR) OTAS-LSM in the imaging of optically cleared mouse brains. **a**, **b** MIP images of dendrites acquired by CM and HR-OTAS-LSM, respectively. Magnified images of the spines (**c**, **d**) and varicosities (**e**, **f**) of dendrites acquired by CM and HR-OTAS-LSM, respectively. Depth color-coded LR (**g**) and HR (**h**) OTAS-LSM images in the end region of the cerebral cortex and magnified images (**i**, **j**) of marked ROIs in **g** and **h**

Figure 5 shows the comparison results of HR-OTAS-LSM with two relevant microscopy techniques, conventional point scanning confocal microscopy (CM) and low-resolution OTAS-LSM, in the imaging of optically cleared mouse brains. CM is a high-resolution 3D imaging technique that operates by raster scanning a laser focus and spatially filtering emission light with a confocal pinhole. Although CM can achieve 3D imaging at sub-micron resolutions akin to HR-OTAS-LSM, it does so at significantly lower image throughputs or in lower image contrasts. The disparity stems from the point scanning approach of CM in contrast to the line scanning one of HR-OTAS-LSM. In HR-OTAS-LSM, emission light is captured from the focused excitation light sheet line by line, permitting pixel signal integration over a greater time span than CM, given the same imaging speed. In the comparison study, a commercial CM equipped with swift galvanometer scanners was employed to capture images of identical brain slice specimens at a comparable image throughput to that of HR-OTAS-LSM by employing a larger voxel size of 0.45 µm × 0.45 µm × 1 µm than the one of HR-OTAS-LSM. Both large FOV images of the same brain slice specimen and magnified images in the selected ROIs of the large FOV images are presented for comparison. Both CM and HR-OTAS-LSM images showed detailed cell structures. In the magnified images of the red ROI in Fig. 5c, d, the spines extended from a single dendrite (yellow arrows) were visualized. In the magnified images of the blue ROI (Fig. 5e, f), thin dendrites with partially bright varicosities were visualized (green arrows). Although both CM and HR-OTAS-LSM images showed similar detailed dendrite structures, CM images suffered from the reduced image contrasts, as the consequence of their shorter pixel dwell time compared to HR-OTAS-LSM images. The pixel dwell times of CM and HR-OTAS-LSM images were 0.1 µs and 19.5 µs, respectively. Given its approximately 200-fold longer pixel dwell time and superior image contrast, HR-OTAS-LSM outperformed CM in either image throughput or contrast.

HR-OTAS-LSM was compared with the low-resolution (LR) OTAS-LSM, and the imaging results are presented in Fig. 5g–j. LR-OTAS-LSM was a previous OTAS-LSM method utilizing air objective lenses in both illumination and imaging arms, and it had image resolutions of approximately 1.5 µm in both the lateral and axial directions [18]. The comparison results encompassed depth color-coded images from the analogous cerebral cortex regions and magnified images of ROIs situated near the cerebral cortex’s periphery. While the LR system primarily visualized relatively large cellular structures due to its limited resolution, the HR system visualized intricate structures at terminal points.

### HR-OTAS-LSM imaging of mouse retina

**Fig. 6:**
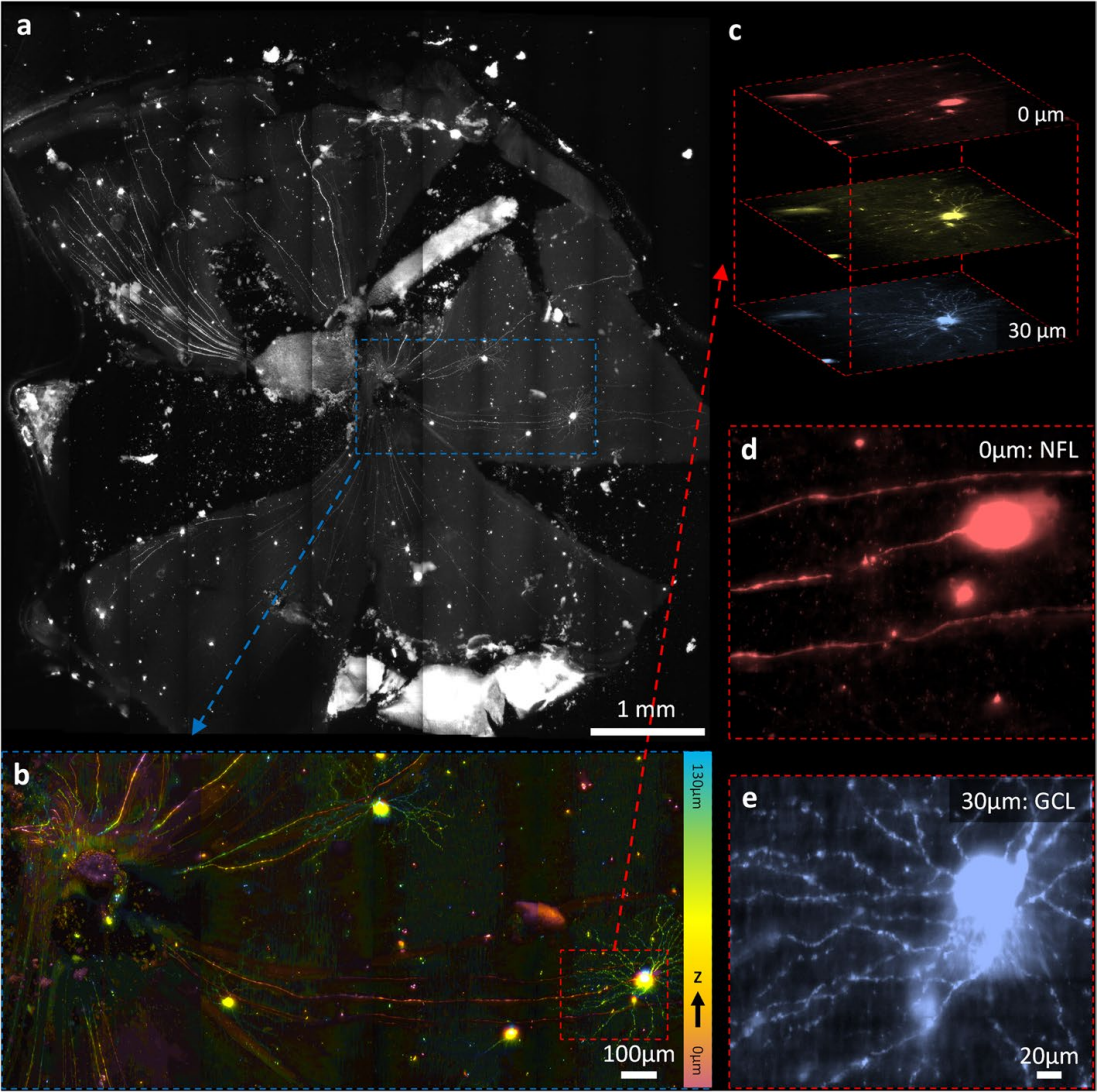
HR-OTAS-LSM images of a Thy1-eYFP mouse retinal flat mount. **a** A large FOV MIP enface image showing the entire mouse retinal flat mount. **b** A magnified depth color-coded image showing a ganglion cell on the right with a long axon extended to the optic nerve head on the left. **c**–**e** Two magnified images at different depths showing the 3D distribution of axons and dendritic trees of the ganglion cells. NFL, nerve fiber layer; GCL, ganglion cell layer

Figure 6 shows the HR-OTAS-LSM images of the mouse retinal flat mount. The entire imaging area was 5 mm × 5 mm in the *x*–*y* plane, and images in various FOVs are presented. The large FOV image was the MIP of 3D images with a 130-µm depth range, and it visualized sparsely distributed YFP-expressing retinal ganglion cells (RGCs) with long axons connected to the optic nerve head. An ROI was selected over a single RGC and magnified in Fig. 6b. The magnified image was depth color-coded down to 30 µm deep from the inner retinal surface, covering both the nerve fiber layer (NFL) and ganglion cell layer (GCL). Both the axons and dendrites were visualized in overlap. An ROI was selected around the cell body, and two MIP images covering different depth ranges were generated to visualize the 3D distribution of axons and dendrites. While the axon from the optic disk was connected to the cell body in the superficial NFL, fine dendrites from the cell body extended radially and formed a dendritic tree structure in the lower GCL [19]. The varicosities of RGC were also identified along the dendrites.

## Discussion

HR-OTAS-LSM was developed for high-throughput and submicron-resolution imaging of optically cleared tissue. HR-OTAS-LSM achieved the high axial resolution by axially sweeping the aberration-corrected, tightly focused light sheet with the DM in the illumination arm and the high lateral resolution by using the high NA immersion objective lens in the imaging arm. The transverse and axial image resolutions were 0.4 and 0.9 µm, respectively. The imaging FOV was approximately 0.5 mm × 0.5 mm in the oblique image plane, and the maximum imaging speed was up to 100 fps with a reduced FOV. HR-OTAS-LSM performance was demonstrated in the imaging of optically cleared mouse brain and transparent retinal flat-mount specimens. Both the gross neuronal network and the fine details of individual neurons were visualized. With high image resolutions in all dimensions, HR-OTAS-LSM might be useful for detecting subtle changes in the neuronal network associated with some diseases or degeneration.

The maximum imaging depth was 1.5 mm due to both the angled imaging arm with respect to the sample surface and the wide and flat front end of the imaging objective lens. The maximal working distance was achieved by tilting the imaging arm to 60° with respect to the sample surface. The maximum imaging speed was currently limited by the camera speed. High-speed cameras could be used for higher imaging speeds. The lateral step size in full-resolution sampling was approximately 0.2 µm. For higher imaging throughput, some level of under-sampling can be used, followed by employing an image restoration algorithm to restore the reduced image quality [20]. No additional image processing algorithm such as deconvolution was used in the current study because most detailed cell structures were resolved without artifacts. However, there were stripe pattern artifacts at high imaging depths due to the absorption and scattering of the illumination light sheet by the sample. These stripe patterns would be suppressed using the recently developed processing algorithms [21].

Various high-resolution high-speed OT-LSM techniques have been reported recently, among which swept confocally aligned planar excitation (SCAPE) microscopy, DaXi microscopy, and hybrid OT-LSM stand out with their impressive capabilities of achieving sub-micron lateral and sub-2 μm axial resolution [3, 13, 14]. Notably, SCAPE and DaXi microscopies belong to the category of single objective (SO)-OT-LSM, while the hybrid OTLSM does to dual objective (DO)-OT-LSM. SO-OT-LSM techniques including SCAPE and DaXi microscopies operate with single-objective lenses, leading to small crossing angles less than 90° between the illumination and detection paths. The small angles limit the axial resolution and affect the illumination and collection efficiency. The hybrid OT-LSM, despite being a DO-OT-LSM system, also has a constrained axial resolution in high-resolution mode due to a relatively small crossing angle of approximately 45° (NODO OT-LSM) between the illumination and detection paths. On the other hand, HR-OTAS-LSM benefits from the orthogonal configuration of illumination and detection paths. This configuration provides the best axial resolution and enables HR-OTAS-LSM to capture cellular structures with improved clarity and precision along the axial direction. Despite the apparent complexity of the system architecture using two separate objective lenses and the liquid prism interface, HR-OTAS-LSM is conceptually similar to conventional LSMs, and the imaging workflow is straightforward. The illumination and imaging arms are modular, and the construction and the alignment can be made with instruction. Fabrication of the liquid prism interface is also a straightforward task with the provided CAD drawing (Additional files 1 and 2). Notably, the system’s high-resolution imaging capabilities are attained with minimal post-processing. Thus, high-resolution images can be efficiently acquired in a high-throughput manner. This simplicity positions HR-OTAS-LSM as a system that can be adopted by biologists with proper instruction manuals, without the need for advanced technical expertise.

## Conclusions

HR-OTAS-LSM was developed for the high-throughput cellular imaging of optically cleared large tissue at submicron resolution. Its lateral and axial resolutions were 0.4 and 0.9 µm, respectively. The throughput was 210 MHz in pixel rate (8.4 mm^3^/h). Its performance was demonstrated by high-throughput and high-resolution visualization of neuronal networks in the mouse brain and retina. HR-OTAS-LSM has the potential for the high-throughput cellular examination of optically cleared tissue specimens.

## Methods

### Aberration correction procedure

**Fig. 7:**
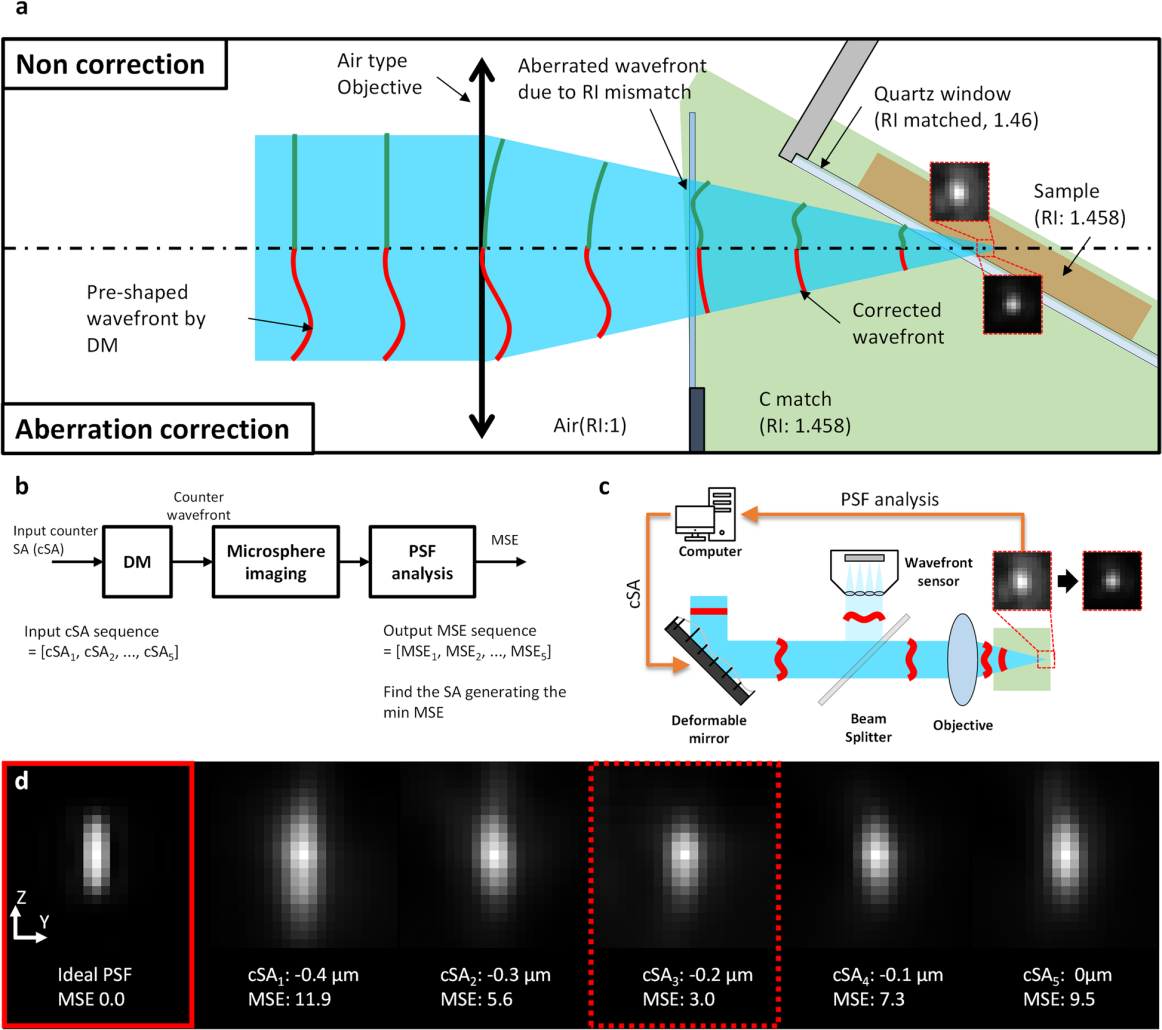
Aberration correction in the Illumination arm. **a** A detailed diagram showing the wavefronts without and with aberration correction caused by RI mismatch. **b**, **c** Schematics showing the aberration correction process. **d** Comparison among the simulated ideal PSF and acquired PSF images under various SA conditions, along with their MSE values relative to the ideal PSF (*n* = 500, *P* < 0.01). DM, deformable mirror; RI, refractive index; SA, spherical aberration; MSE, mean squared error

Optical aberration in the illumination arm was corrected during the calibration phase before initiating the sample imaging. The major aberration was SA, arising from the RI mismatch between the air and the immersion liquid in the path of excitation light. To estimate the level of SA in the system, an initial assessment was performed through simulation. This simulation utilized both the geometric information of light rays as they traveled from the air objective lens to the focal point within the sample and RI information of the media. A detailed description of the simulation procedure can be found in the low-resolution OTAS-LSM paper [18]. The system SA was estimated to be + 0.2 μm. To correct the aberration, DM was employed to generate a counter SA with a negative value. Figure 7 illustrates the correction process. Without correction, as depicted in Fig. 7a, the ideal flat wavefront before the air objective lens was distorted as it traversed the RI matching solution and the sample. With correction, the wavefront before the objective lens was pre-shaped by the DM and subsequently became an ideal state in the RI matching solution. Figure 7b, c delineates the precise aberration correction steps. To gauge the effectiveness of this correction, images of microspheres as point sources were captured, and the resulting PSF was obtained via averaging. The mean square error (MSE) value, quantifying the deviation between the measured PSF and the ideal PSF, served as the objective metric. The ideal PSF was constructed by convolution with the microsphere. Recognizing that the simulation predicting system aberrations could entail errors, a sequence of five counter SA values was generated around the initial counter SA value (− 0.2 μm) with increments of 0.1 μm. The MSE values corresponding to the counter SA levels were then measured. Ultimately, the counter SA value yielding the lowest MSE value was selected as the optimal correction. This optimal counter SA value was applied to the DM for sample imaging. Throughout the aberration correction process, the LabVIEW SDK (Software Development Kit, Thorlabs) was utilized to control the DM. The coefficients of Zernike polynomials, ranging from z4 to z15, were provided as input parameters for the correction procedure.

### Sample preparation

After characterization, HR-OTAS-LSM was applied to optically cleared brain slices and transparent retina specimens of Thy1-eYFP mice, ex vivo. All animal procedures in this study were approved by the Institutional Animal Care and Use Committee (POSTECH-2019–0061). To prepare optically cleared mouse brain specimens, mice were euthanized with the intravenous injection of the Zoletil and Rompun mixture in a 1:1 ratio, and intracardiac perfusion was performed by intravenously injecting a PBS and 4% paraformaldehyde (PFA) mixture in a 1:1 ratio using a peristaltic pump. The fixed mouse brain was sliced coronally in 2–3 mm thickness. The brain slices were incubated in RI matching solution for 2 h at 36 °C for tissue clearing. For the retina specimens, the mice were sacrificed by cervical dislocation. The enucleated mouse eyes were washed with cold 1 × PBS and fixed in 4% PFA for 1 h at room temperature. After fixation, any remnant fat, muscle, episcleral, or optic nerve tissues were removed. The cornea and anterior sclera were then removed through a surgical incision along the equator, and the crystalline lens was subsequently removed. The retina and choroid cup were flattened with multiple meridional incisions. The retinal flat mounts were imaged without optical clearing.

### HR-OTAS-LSM imaging

The tissue specimens were mounted on the sample holder and imaged in 3D through stepwise translation along the *x*, *y*, and *z* directions. Typically, long image strips in the *x* direction were acquired with stepwise translation first at 100 fps. Multiple image strips were acquired with translation in the orthogonal *y* or *z* direction. The raw image strip was sheared at 30° with respect to the sample surface, and a custom image processing algorithm was used to transform the image data in the *xyz* coordinate. Planar images in the enface (*xy*) and cross-sectional (*xz*) planes could be accessed from the transformed 3D data. Mosaic images were generated by joining the image strips with the manual estimation of overlaps and relative coordinates in 3D rendering software (Matlab, Mathworks). For the mouse brain and flat-mount retina specimens, 28 and 12 image strips in total were acquired with translation along the *y* direction to cover the entire area, and the imaging times were approximately 2.5 and 0.5 h in total, respectively. The mouse brain was captured at a speed of 50 fps with a 20-ms exposure time to use the full ROI of the sensor, and flat-mounted retina specimens were recorded at a rate of 100 fps with a 10-ms exposure time. The final volumetric mosaic images of the mouse brain and retina specimens were 13 × 7.5 × 0.23 mm and 5 × 5 × 0.15 mm in *xyz* dimensions, respectively. The mouse brain specimens were also imaged in depth with axial translation. The 3D image data covering 1.5 mm in thickness were generated by joining 9 image strips at different depths. All images were presented without any additional post-processing, such as deconvolution.

### Confocal microscopy imaging for comparison with HR-OTAS-LSM imaging

HR-OTAS-LSM was compared with CM in the mouse brain. Confocal imaging was conducted using a commercial system (SP-5, Leica). A multi-immersion × 25 objective lens (HC FLUOAR L 25x/0.95 W, Leica) was used for imaging in the RI matching solution. The imaging FOV was 490 µm × 490 µm comprising 1024 × 1024 pixels. Volumetric images were acquired with an axial step size of 1 µm and an imaging speed of 0.4 fps at a 400-Hz scanner speed.

### Supplementary Information

**Additional file 1.** CAD drawing of customized Liquid prism.

**Additional file 2.** SOLIDWORKS (Dassault Systèmes) part file of customized Liquid prism.

## Data Availability

A complete computer-aided design (CAD) model of the custom liquid prism is detailed in the additional files (Additional file 1 CAD drawing_Liquid prism, Additional file 2 Liquid prism.SLDPRT). All data generated or analyzed during this study are included in this published article and its supplementary information files.
