# Supplementary figures and images for: High-resolution open-top axially swept light sheet microscopy
Source: BMC Biol. 2023 Nov 8;21:248. doi: 10.1186/s12915-023-01747-3 (PMC10634022; doi:10.1186/s12915-023-01747-3)

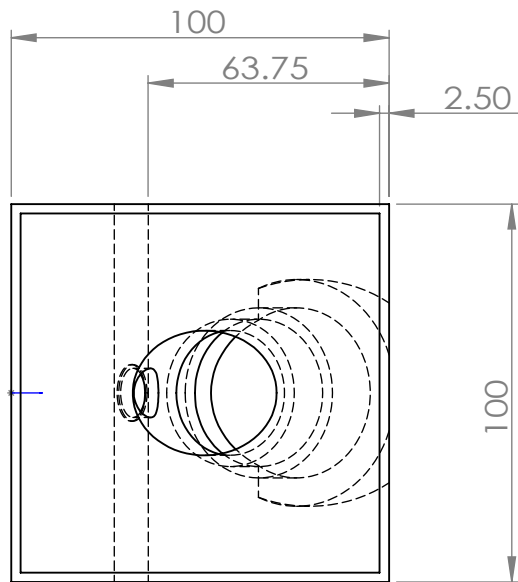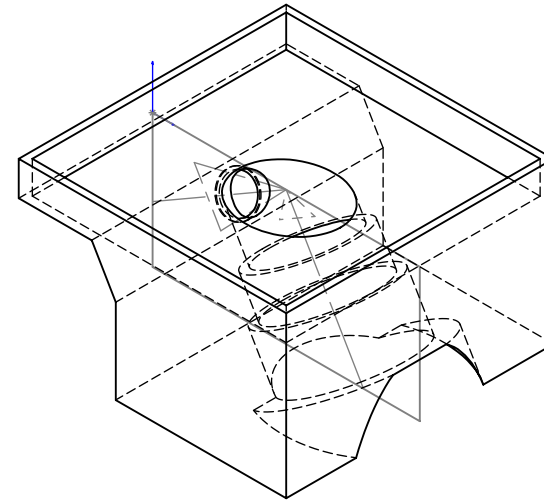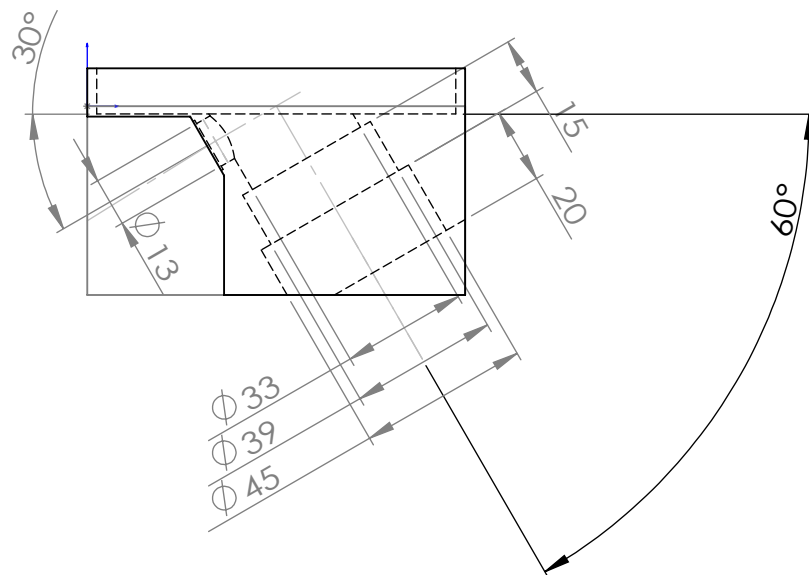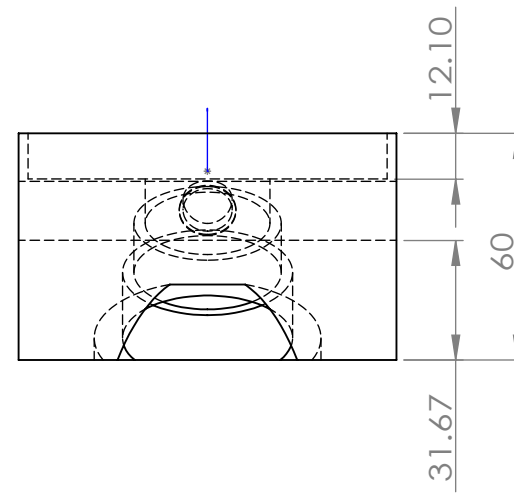

Supplement: Supplementary file 1 — Additional file 1. CAD drawing of customized Liquid prism. [file 12915_2023_1747_MOESM1_ESM.pdf]
